# Maternal HCV infection is associated with intrauterine fetal growth disturbance: A meta-analysis of observational studies: Erratum

**DOI:** 10.1097/01.md.0000503452.53226.6c

**Published:** 2016-10-07

In the article “Maternal HCV infection is associated with intrauterine fetal growth disturbance: A meta-analysis of observational studies”,^[1]^ which appeared in Volume 95, Issue 35 of *Medicine*, some affiliation information was given incorrectly. The article has since been corrected online.
